# Supplementary material for: Liquid-liquid phase separation and extracellular multivalent interactions in the tale of galectin-3
Source: Nat Commun. 2020 Mar 6;11:1229. doi: 10.1038/s41467-020-15007-3 (PMC7060198; doi:10.1038/s41467-020-15007-3)
Supplement: Supplementary file 1 — Supplementary Information [file 41467_2020_15007_MOESM1_ESM.pdf]

Supplementary Information

Liquid-liquid phase separation and extracellular multivalent interactions in the tale of galectin-3

Y.-P. Chiu, Y.-C. Sun et al.

Supplementary Table 1. O.D$_{600}$ (A.U.) for Fig. 1h.

		Galectin-3 (μM)							
		5	10	15	20	25	30	35	40
	0.2	0.01	0.196	0.523	0.969	1.361	1.703	2.053	2.98
LPS	0.1	0.002	0.275	0.592	0.812	1.069	1.334	1.375	1.526
(mg/ml)	0.05	0.02	0.204	0.317	0.465	0.512	0.597	0.680	0.730
	0.0	0	0	0	0	0	0	0	0

Supplementary Table 2. O.D$_{600}$ (A.U.) for Fig. 1i.

		Triplicates of O.D measurement				
		#1	#2	#3	Ave	SD
	5	0.013	0.010	0.007	0.010	0.003
Galectin-3	10	0.128	0.268	0.192	0.196	0.070
(μM)	20	1.092	0.835	0.980	0.969	0.129
	40	2.927	2.978	3.046	2.984	0.060

Supplementary Table 3. O.D$_{600}$ for Fig. 5a.

		Triplicates of O.D measurement				
		#1	#2	#3	Ave	SD
	wt-FL	2.905	3.086	3.034	3.008	0.093
	NTD	0.029	0.061	0.023	0.038	0.020
Construct	CRD	0.039	0.003	0.023	0.022	0.018
(40 μM)	W/G-FL	2.172	1.938	1.841	1.984	0.170
	Y/G-FL	0.008	0.004	0.014	0.009	0.005
	WY/G-FL	0.005	0.003	0.006	0.008	0.002

Supplementary Table 4. UniProt entries for the sequences shown in Fig. 3a

Species	Entry	Entry name
Homo sapiens (Human)	P17931	LEG3_HUMAN
Oryctolagus cuniculus (Rabbit)	P47845	LEG3_RABIT
Rattus norvegicus (Rat)	P08699	LEG3_RAT
Canis lupus familiaris (Dog)	P38486	LEG3_CANLF
Sus scrofa (Pig)	A3EX84	A3EX84_PIG
Bos taurus (Bovine)	A6QLZ0	A6QLZ0_BOVIN
Gallus gallus (Chicken)	A4GTP0	A4GTP0_CHICK
Xenopus laevis (frog)	Q7ZSY1	Q7ZSY1_XENLA
Danio rerio (Zebrafish)	F1QSL3	F1QSL3_DANRE

Supplementary Table 5. Primers used in this study

Construct	Template	Primer Sequence
NTD-WY/G	pHD-Gal3-WY/G	Fw: 5' CTGGGTAACTCGAGCACCAC 3'
		Rv: 5' AGTTACCCAGCAGGGGC 3'
NTD-Y/G	pHD-Gal3-NTD-	Fw: 5' CCTGGCGCATGGGGGAACCAGCCTGCT 3'
	WY/G	Rv: 5' TGCGCCAGGCCATCCTTGAGGGTTTGG 3'
NTD-W/G	pHD-Gal3-NTD	Fw: 5' CCTGGCGCAGGCGGGAACCAGCCTGCT 3'
		Rv: 5' TGCGCCAGGGCCTCCTTGAGGGTTTGG 3'
NTD-GFP	pHD-Gal3-NTD	Fw: 5' CTAGGTACCATGGGATCCAAAGGAGAAGAG 3'
		Rv: 5' CTAGGTACCCCCAGCAGGGGCGCCATA 3'
Y/G	pHD-Gal3-WY/G	Fw: 5' CCTGGCGCATGGGGGAACCAGCCTGCT 3'
		Rv: 5' TGCGCCAGGCCATCCTTGAGGGTTTGG 3'
W/G	pHD-Gal3-wt	Fw: 5' CCTGGCGCAGGCGGGAACCAGCCTGCT 3'
		Rv: 5' TGCGCCAGGGCCTCCTTGAGGGTTTGG 3'

Supplementary Note 1. Estimate of the local galectin-3 concentration on the surface of the micelles

The radius of gyration of galectin-3 CRD is about 15 Å. The maximum distance between a pair of atoms in the CRD is around 35 Å. Two galectin-3 molecules bind either on the same LPS molecule or adjacent LPS molecules on a micelle, 50 Å × 50 Å × 100 Å is an overestimate of the space taken up by two CRDs in close proximity. A rough estimate of the local concentration is therefore:

$$\frac{\frac{2}{6\times10^{23}}(\text{mole})}{50(\text{Å})\times50(\text{Å})\times100(\text{Å})\times10^{-27}(\frac{\text{L}}{\text{Å}^3})} = \frac{1}{75}(\text{M}) \approx 13.3(\text{mM})$$

Supplementary Note 2. The synthesized cDNA of the WY/G construct.

ATGGCAGACAATTTTTCGCTCCATGATGCGTTATCTGGGTCTGGAAACCCAAACCCT
CAAGGAGGCCCTGGCGCAGGCGGGAACCAGCCTGCTGGGGCAGGGGGCGGCCCAGGG
GCTTCCGGCCCTGGGGCCGGCCCCGGGCAGGCACCCCCAGGGGCTGGCCCTGGACAG
GCACCTCCAGGCGCCGGCCCTGGAGCACCTGGAGCTGGCCCCGGAGCACCTGCACCT
GGAGTCGGCCCAGGGCCACCCAGCGGCCCTGGGGCCGGCCCATCTTCTGGACAGCCA
AGTGCCACCGGAGCCGGCCCTGCCACTGGCCCCGGCGGCGCCCCTGCTGGGCCACTG
ATTGTGCCTTATAACCTGCCTTTGCCTGGGGGAGTGGTGCCTCGCATGCTGATAACA
ATTCTGGGCACGGTGAAGCCCAATGCAAACAGAATTGCTTTAGATTTCCAAAGAGGG
AATGATGTTGCCTTCCACTTTAACCCACGCTTCAATGAGAACAACAGGAGAGTCATT
GTTTGCAATACAAAGCTGGATAATAACTGGGGAAGGGAAGAAAGACAGTCGGTTTTC
CCATTTGAAAGTGGGAAACCATTCAAAATACAAGTACTGGTTGAACCTGACCACTTC
AAGGTTGCAGTGAATGATGCTCACTTGTTGCAGTACAATCATCGGGTTAAAAAACTC
AATGAAATCAGCAAACTGGGAATTTCTGGTGACATAGACCTCACCAGTGCTTCATAT
ACCATGATATAA

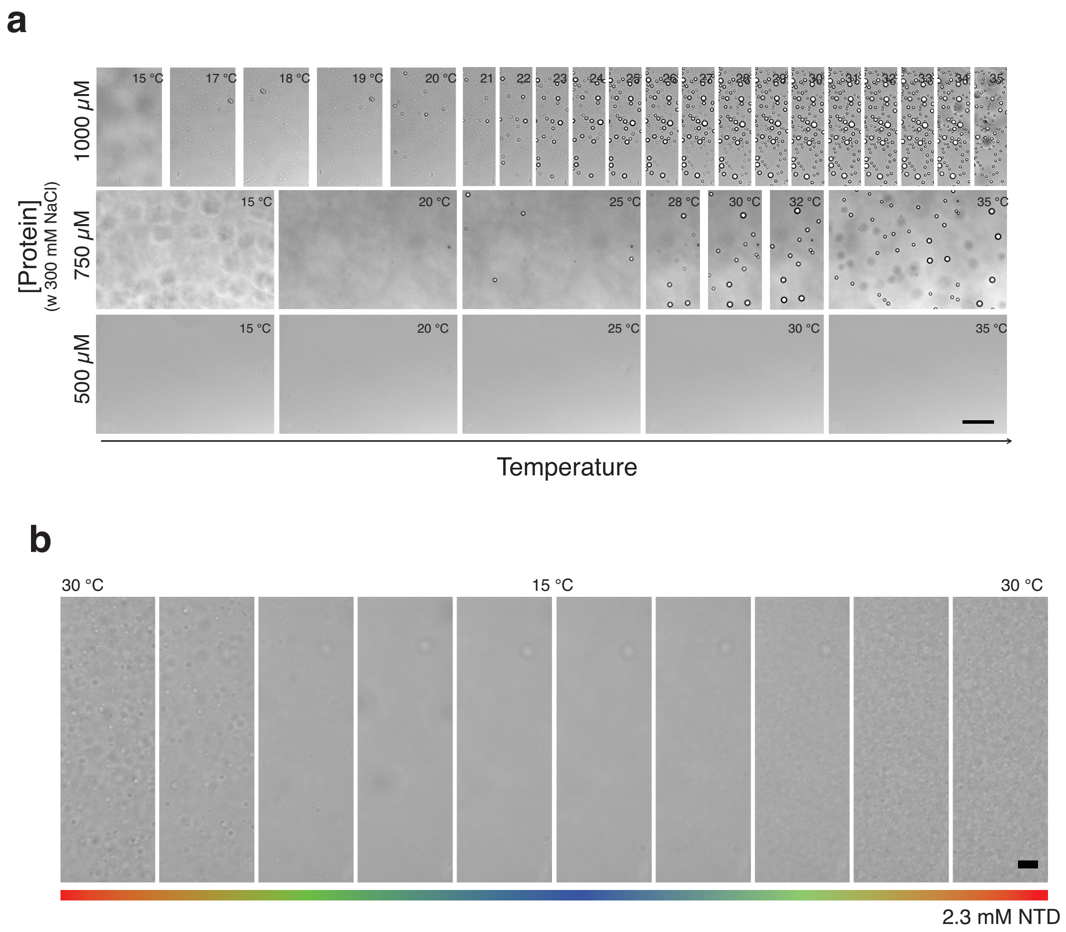

Supplementary Figure 1. (a)The effects on condensate formation of protein concentration and temperature (at a fixed salt concentration of 300 mM). Scale bar: 50 μm. (b) The LLPS of 2.3 mM NTD in the absence of NaCl. Scale bar: 10 μm Experiments were performed at least three times for each protein sample.

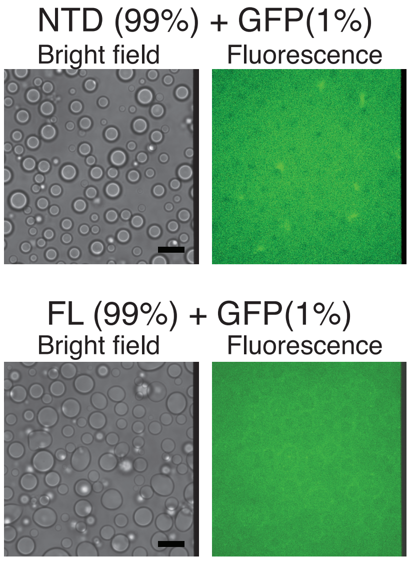

Supplementary Figure 2. The NTD and full-length samples under the conditions of the two-phase regime mixed with 1 % of GFP. GFP has no preference to enter the condensate in both instances (see Fig. 2c, 2h of GFP-tagged NTD for comparison). Scale bar: 50 μm. These control experiments were performed once.

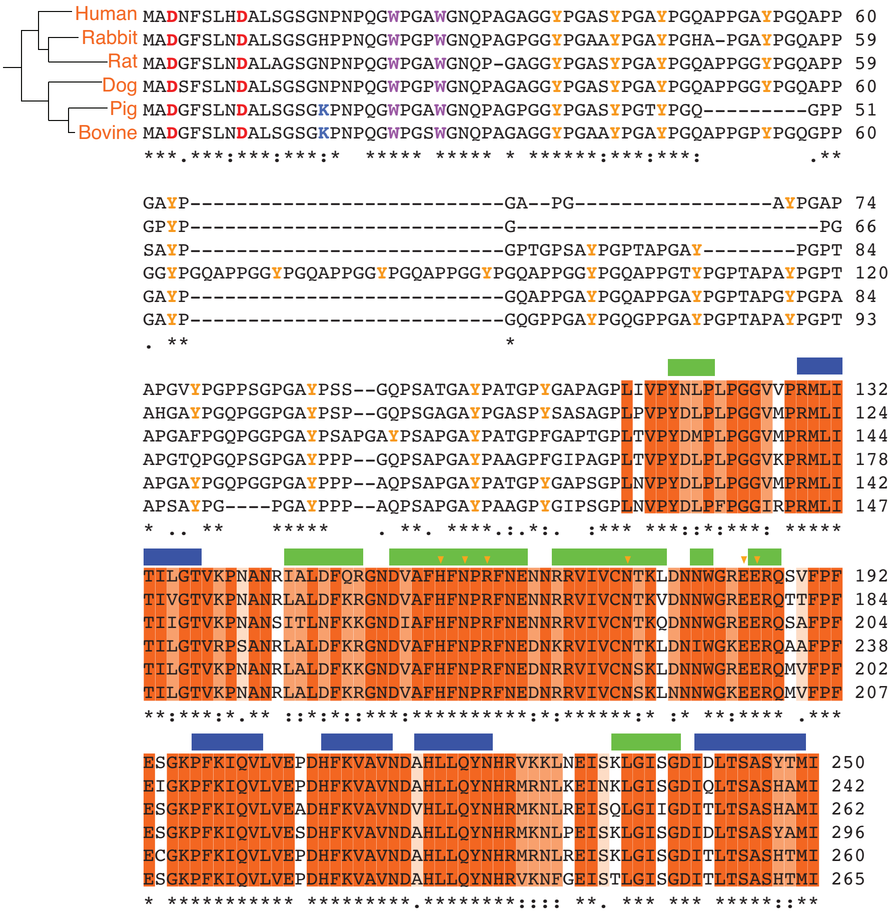

Supplementary Figure 3. Comparison of representative mammalian galectin-3 amino-acid sequences. The residues in the carbohydrate-recognition domain are highlighted with dark, medium, or light orange depending on the level of conservation. Negatively charged, positively charged, tryptophan, and tyrosine residues in the N-terminal domain are shown in red, blue, purple and yellow, respectively.

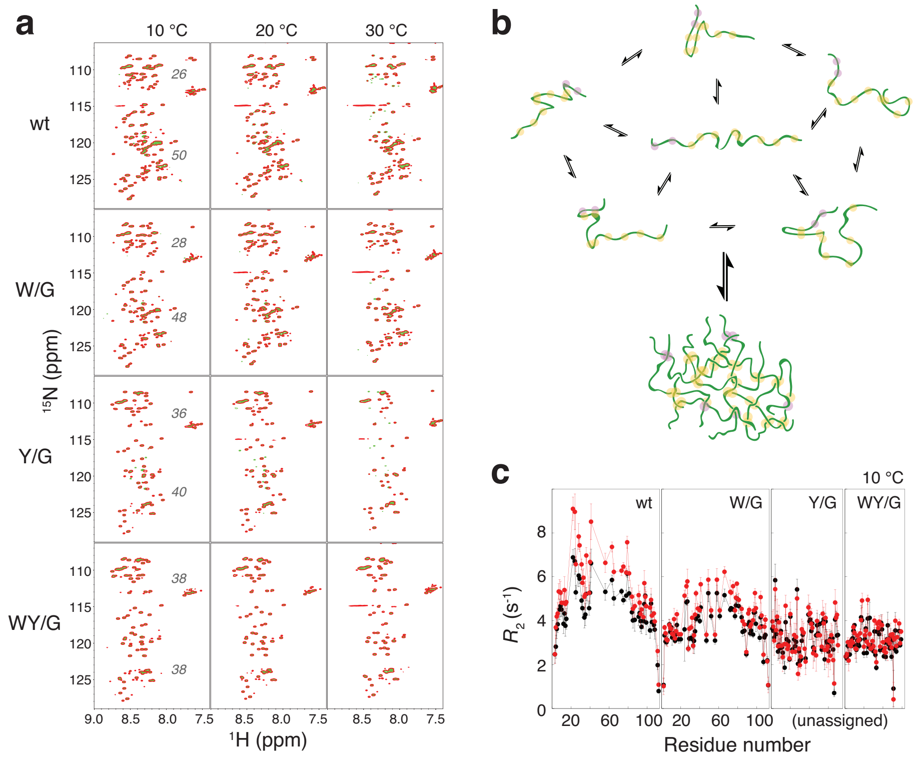

Supplementary Figure 4. NMR studies of N-terminal domain (NTD) constructs of galectin-3. (a) HSQC spectra of 40 (green) or 400 µM (red) samples of the different constructs at different temperatures. The expected numbers of peaks from glycine (upper number) and other residues (lower number) are shown in the spectrum recorded at 10°C for each construct. There are more peaks than expected for the wild type because the conformation changes at equilibrium occur in the slow exchange regime (vs the NMR timescale). This exchange probably involves the aromatic residues because when they are all removed, most of the extra peaks disappear (WY/G spectra). (b) Schematic representation of inter- and intramolecular NTD interactions through aromatic residues. (c) Transverse relaxation rate constants (R_2) of 40 µM (black) and 400 µM (red) samples of the different constructs at 10°C. The overall reduction in R_2 vs the wild type for the constructs without aromatic residues indicates that inter- and intramolecular conformational exchanges are abolished.

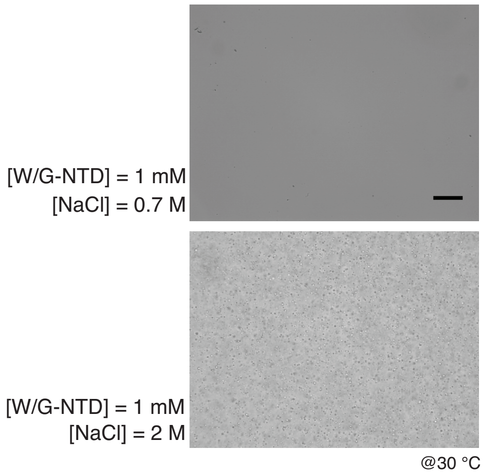

Supplementary Figure 5. The micrographs of 1 mM W/G-NTD construct. The 1 mM wild-type sample phase-separated when the NaCl concentration is above 0.15 M, but even with 0.7 M salt, W/G-NTD remains in the one-phase regime. When the NaCl concentration is 2 M, the W/G-NTD construct shows condensate probably due to the contribution of tyrosines because there is no condensate for the WY/G-NTD construct under the same condition (Fig. 3e). Scale bar: 50 µm. Experiments were performed at least three times for each protein sample.

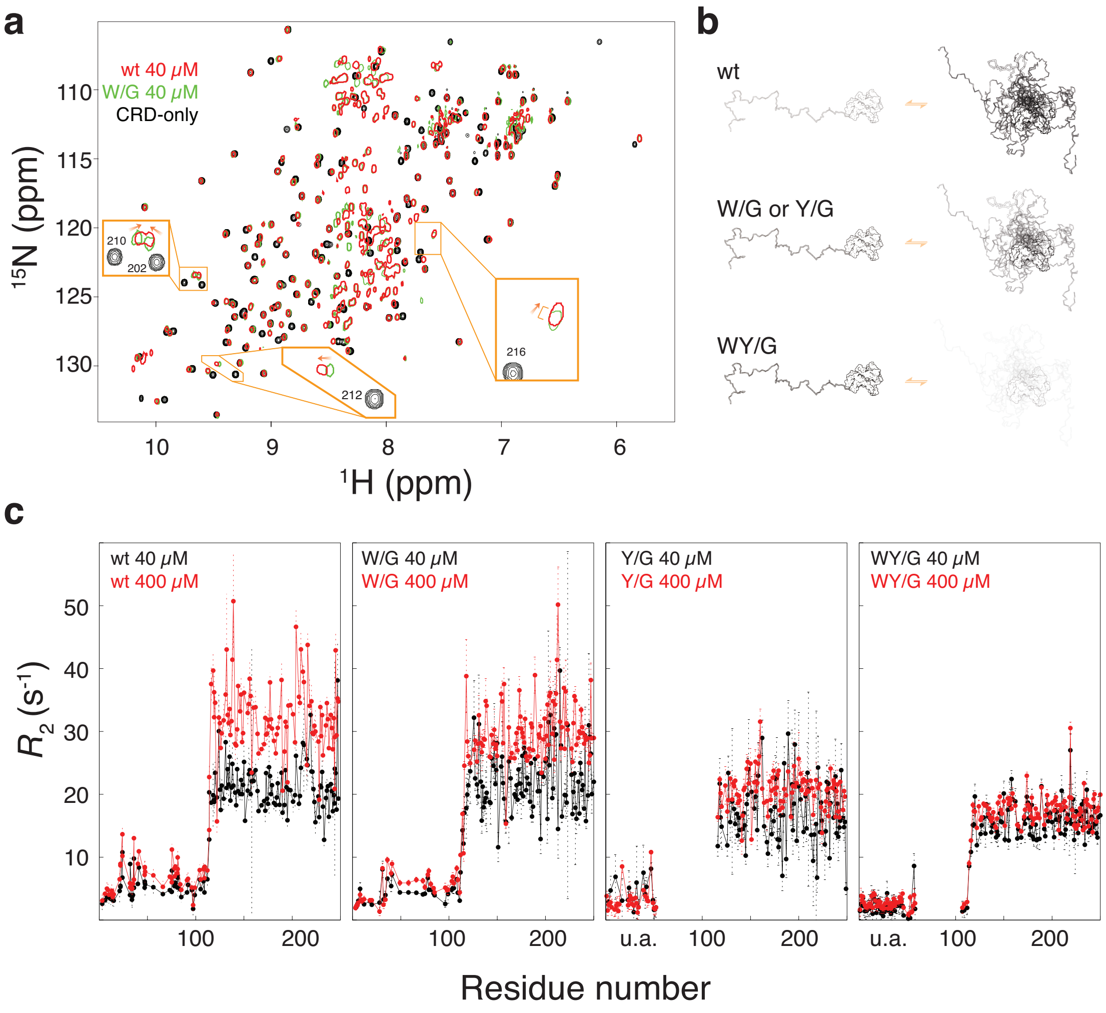

Supplementary Figure 6. NMR studies of full-length galectin-3 constructs. (a) Comparison of HSQC spectra of 40 μM samples of the wild type (red) and of the W/G (green) construct with that of a carbohydrate recognition domain (CRD)-only construct. The changes in chemical shift (orange arrows) indicate more N-terminal domain (NTD)-CRD interactions in the wild type than in the W/G mutant. These interactions are abolished in the WY/G construct (see the main text). (b) Illustration of the proposed equilibrium population distributions of the wild type and the two constructs between the fully extended conformation and one with NTD-CRD interactions. The darker image represents the higher population. (c) Transverse relaxation rate constants (R_2) for wild type galectin and the three constructs at 40 μM (black) and 400μM (red). As in Fig. S4c, the overall R_2 decreases when the aromatic residues are replaced with glycine. These results reinforce the conclusion that protein dynamics increase as inter- and intramolecular interactions become rarer.

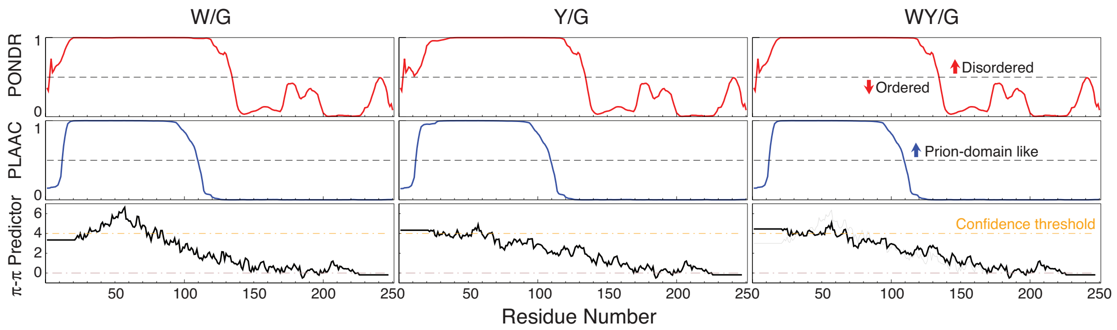

Supplementary Figure 7. Sequence analyses of aromatic-substituted mutants of the level of structural disorder, similarity to prion-like proteins, and the propensity to form π- π interactions

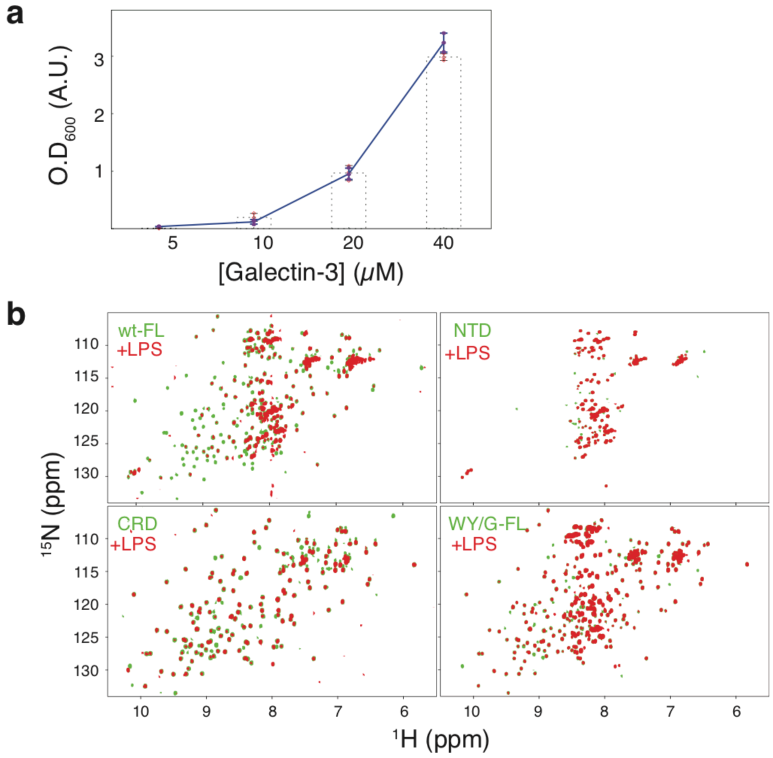

Supplementary Figure 8. (a) Turbidity ($O.D_{600nm}$) measured under different galectin-3 concentration in the presence of 300 mM NaCl (blue lines, n=3 independent samples; data are presented as mean values +/- SD) in comparison to without NaCl (dashed black bars, also see Fig. 1i). (b) NMR studies of lipopolysaccharide (LPS)/galectin-3 mixtures. HSQC spectra of 40 μM samples in the absence (green) or in the presence of LPS micelles (red).

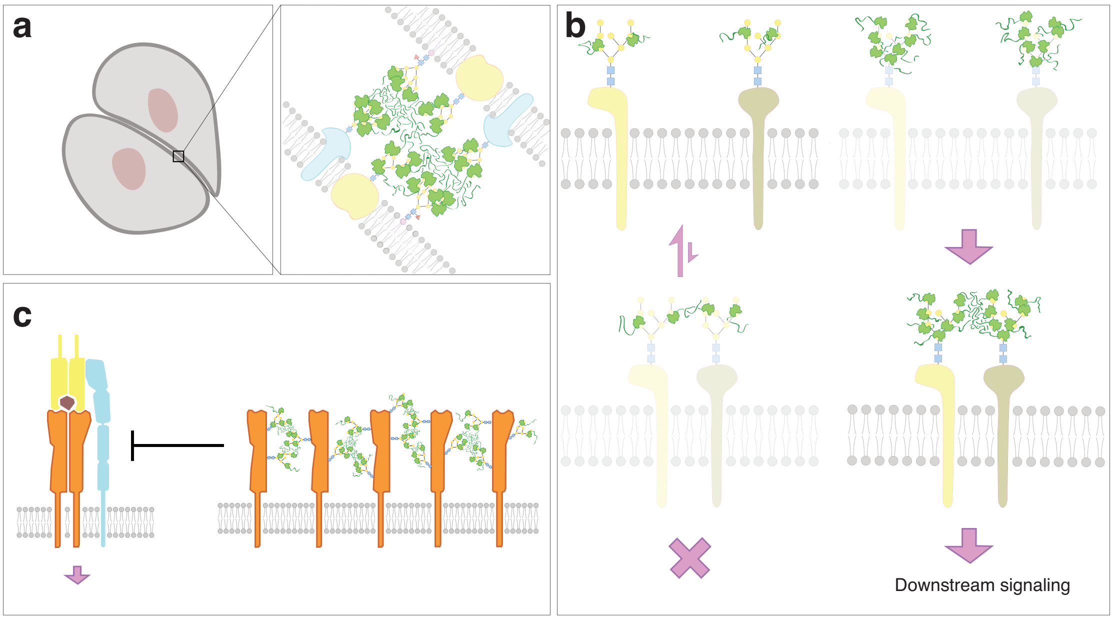

Supplementary Figure 9. Schematic illustrations of how multiple weak interactions can collectively mediate galectin-3 functions: (a) cell-cell adhesion, (b) signal activation, and (c) signal inhibition.
