# Supplementary material for: Liquid-liquid phase separation and extracellular multivalent interactions in the tale of galectin-3
Source: Nat Commun. 2020 Mar 6;11:1229. doi: 10.1038/s41467-020-15007-3 (PMC7060198; doi:10.1038/s41467-020-15007-3)
Supplement: Supplementary file 3 — Description of Additional Supplementary Information [file 41467_2020_15007_MOESM3_ESM.pdf]

Description of Additional Supplementary Files

File Name: Supplementary Movie 1
Description: A sample of 1 mM N-terminal domain of galectin-3 with 150 mM NaCl condensing at 35 °C and dissolving at 15 °C reversibly.

File Name: Supplementary Movie 2
Description: Monitoring the fusion of condensates from a sample of 1 mM N-terminal domain of galectin-3 with 150 mM NaCl at 35 °C.

File Name: Supplementary Movie 3
Description: A 500 µM full-length galectin-3 sample with 800 mM NaCl condensing reversibly (30–15–30 °C).
